# Tear fluid calcitonin gene-related peptide (CGRP) is elevated during spontaneous migraine attacks – results from a pilot study

**DOI:** 10.1186/s10194-025-02255-1

**Published:** 2025-12-25

**Authors:** Katharina Kamm, Annika Brandi-Dohrn, Andreas Straube, Stefanie Förderreuther, Ruth Ruscheweyh

**Affiliations:** Department of Neurology, University Hospital, LMU Munich, Marchioninistr. 15, 81377 Munich, Germany

**Keywords:** Migraine, CGRP, Headache, Tear fluid

## Abstract

**Background:**

Calcitonin gene-related peptide (CGRP) plays an important role in the pathophysiology of migraine. The peptide is elevated during interictal and ictal migraine and it might be a biomarker for the disease. However, CGRP detection in blood is limited by dilution and rapid degradation. Therefore, we investigated tear fluid CGRP during spontaneous migraine attacks.

**Methods:**

Episodic migraine patients were investigated at two study days. At study day 1, tear fluid was sampled interictally (‘interictal’) and a thorough migraine history was conducted. At study day 2, participants were investigated during a spontaneous migraine attack. Participants were asked to call the study team when experiencing a migraine attack and to present to the outpatient headache center. After arrival, tear fluid was sampled and headache characteristics were assessed. Tear fluid CGRP levels at maximum headache intensity (‘headache’) and after headache improvement (‘post headache’) were analyzed using a commercial CGRP ELISA.

**Results:**

14 female migraine patients (28.4 ± 9.3 years) were included in the analysis. At the time of maximum headache, tear fluid CGRP levels were significantly higher compared to CGRP levels at baseline and after headache resolution (‘interictal’: 1.89 ± 1.68 ng/ml, headache: 2.34 ± 2.20 ng/ml, post headache: 1.23 ± 0.80 ng/ml; *p* = 0.004). The rise of tear fluid CGRP levels was significantly higher if time since headache onset was shorter (0–3 h: +1.80 ± 1.18 ng/ml, 3–6 h: +0.13 ± 0.93 ng/ml, > 6 h: -1.15 ± 1.63 ng/ml; *p* = 0.017).

**Conclusion:**

Tear fluid CGRP levels are elevated during spontaneous migraine attacks, suggesting that the detection of CGRP in tear fluid is valid and might be a migraine biomarker in future.

## Introduction

Recurrent moderate-to-severe headache attacks characterize the primary headache disorder migraine [1]. The clinical course of a migraine attack is well described, however, the pathophysiology is incompletely understood [2, 3].

The neuropeptide calcitonin gene-related peptide (CGRP) is essential for migraine pathophysiology [4]. Upon activation of the trigeminal system, the peptide is released from peripheral trigeminal endings. CGRP binds to trigeminal afferents and the pain signal is transferred to the trigeminal ganglion and higher-order neurons in the brain stem and cerebrum [5]. Earlier studies have shown that CGRP is elevated interictally and rises even further during spontaneous and experimental migraine attacks. In these studies, CGRP was predominantly detected in blood. Due to the results of these studies CGRP was proposed as a diagnostic and therapeutic biomarker for migraine [6–8]. However, the detection in blood was inconsistent, so other body fluids were investigated and it was shown that CGRP can be detected in saliva and tear fluid [9–12]. Advantages of these detection sites are higher peptide concentrations due to the direct innervation of the eye and salivary glands by the trigeminal nerve [13]. Previously, we showed that tear fluid CGRP is elevated in interictal migraine and in experimental migraine attacks [11, 12]. In the present study, tear fluid CGRP was investigated over the course of a spontaneous migraine attack to further validate its potential use as a diagnostic biomarker.

## Methods

### Participants

Participants were recruited at our outpatient headache center and by advertisements at the University Hospital of the Ludwig-Maximilians-University Munich. The study was conducted in accordance with the Declaration of Helsinki and was approved by the LMU ethics committee (18–827). All participants gave written informed consent.

Episodic migraine patients according to the ICHD-3 criteria aged between 18 and 65 years were included [1]. The use of a stable preventive medication for migraine was allowed. Other headache disorders, wearing contact lenses at study days, pre-existing neurological, ophthalmological or severe medical or psychiatric conditions were exclusion criteria.

Further, subjects with known arterial hypertension or a blood pressure of > 140/90mmHg at one of the study days as well as breast-feeding or pregnant women were excluded.

### Study procedure

The study was conducted between 10/2020 and 03/2022 at the outpatient headache center of the LMU university hospital. Participants were investigated at two separate study days. At study day 1, participants were investigated interictally, meaning migraine patients were free of headache and acute medication for 48 h prior to and after the study day. A thorough interview concerning medical and migraine history was conducted, followed by tear fluid sampling of both eyes as earlier described [11, 12]. At study day 2, participants were investigated during a spontaneous migraine attack. Participants were asked to call the study team upon onset of a migraine attack and to present to the outpatient headache center as soon as possible. After arrival, tear fluid was sampled from both eyes. Subsequently, tear fluid was collected regularly. Prior to each sampling, patients filled a headache questionnaire, rating headache intensity on a numerical rating scale (NRS) from 0 to 10 and indicating the presence of migraine symptoms. Patients were allowed to use their regular abortive medication (AM) as needed. If so, an additional sample was collected before intake of the medication. One to two hours after substantial improvement of headache, patients were discharged. On average, 4 ± 1 tear fluid samples were collected per patient.

Interictal tear fluid CGRP levels from day 1 (‘baseline’) were compared to CGRP levels at maximum headache intensity (‘headache’) and to those after headache improvement (‘post headache’). Further, group comparisons were performed based on headache duration at the time of the ‘headache’ sampling and on the type of abortive medication.

Tear fluid CGRP levels were determined using a commercial CGRP ELISA kit (Cusabio^®^, Wuhan, China; detection range: 1.56–100 pg/ml, minimal detectable dose: 0.39 pg/ml, intra-assay precision: < 8%, inter-assay precision: < 10%), following manufacturer’s instructions. Duplicate measurements were performed for each sample. Absorbance values were read using a spectrometer (PerkinElmer, USA) and CGRP concentrations were determined from calibration curves using a 4PL fitting. The final CGRP concentration of each sample was calculated as the average of the two measurements. Interictal tear fluid CGRP levels did not differ between the right and left eye of migraine patients (right eye: 1.87 ± 1.98 ng/ml, left eye: 1.91 ± 1.72 ng/ml; z = 0.157 *p* = 0.875), therefore values from both eyes were pooled.

### Statistics

Data is presented as mean ± standard deviation unless stated otherwise. According to the Shapiro-Wilk-Test some data was non-normally distributed. Therefore, non-parametric tests were used. For comparison of headache intensity and CGRP levels at different points in time, Friedman test was used followed by post-hoc Dunn-Bonferroni tests if appropriate. For comparison of headache intensity or CGRP levels between groups (headache duration groups, type of acute medication groups), Mann-Whitney-U-test or Kruskal-Wallis test was used. Statistical analysis was performed with SPSS 29 (IBM Corp., Armonk, NY, USA). Significance was accepted at *p* < 0.05 (two-tailed).

## Results

82 migraine patients participated in study day 1. Of these, 16 patients contacted the study team to report a migraine attack. Two participants were excluded because tear fluid sampling was not successful at one of the 3 time points, leaving 14 patients (28.4 ± 9.3 years, all female) for the present analysis (see Table 1 for patient characteristics).

**Table 1:** Patient characteristics

	EM patients
**n (% f)**	14 (100.0%)
**Age (in years)**	28.4 ± 9.3
**Monthly headache days (MHD)**	7.7 ± 3.1
**Monthly migraine days (MMD)**	3.7 ± 2.4
**Headache intensity (0–10)**	6.3 ± 1.9
**Time since migraine onset (in years)**	14.1 ± 8.9
Abortive medication*	14 (100.0%)
*Triptan*	4 (28.6%)
*NSAID*	8 (57.1%)
*Triptan + NSAID*	2 (14.3%)
**Prophylactic medication**	2 (14.3%)
*Amitriptyline*	2 (14.3%)

*Patients were asked about their usual intake of acute medication. EM, episodic migraine

Patients arrived at the outpatient headache center 4.2 ± 2.9 (1.3 to 11.0) hours after attack onset. During the course of the attack, they reported a maximum headache intensity of 5.7 $\:\pm\:$ 2.1 on the NRS. In 9 patients (64.3%), the headache fulfilled ICHD-3 migraine criteria. All patients eventually took abortive medication, on average 6.4 ± 2.9 (2.8 to 13.5) hours after attack onset, followed by improvement of headache to 2.3 ± 1.8 on the NRS.

**Fig. 1:**
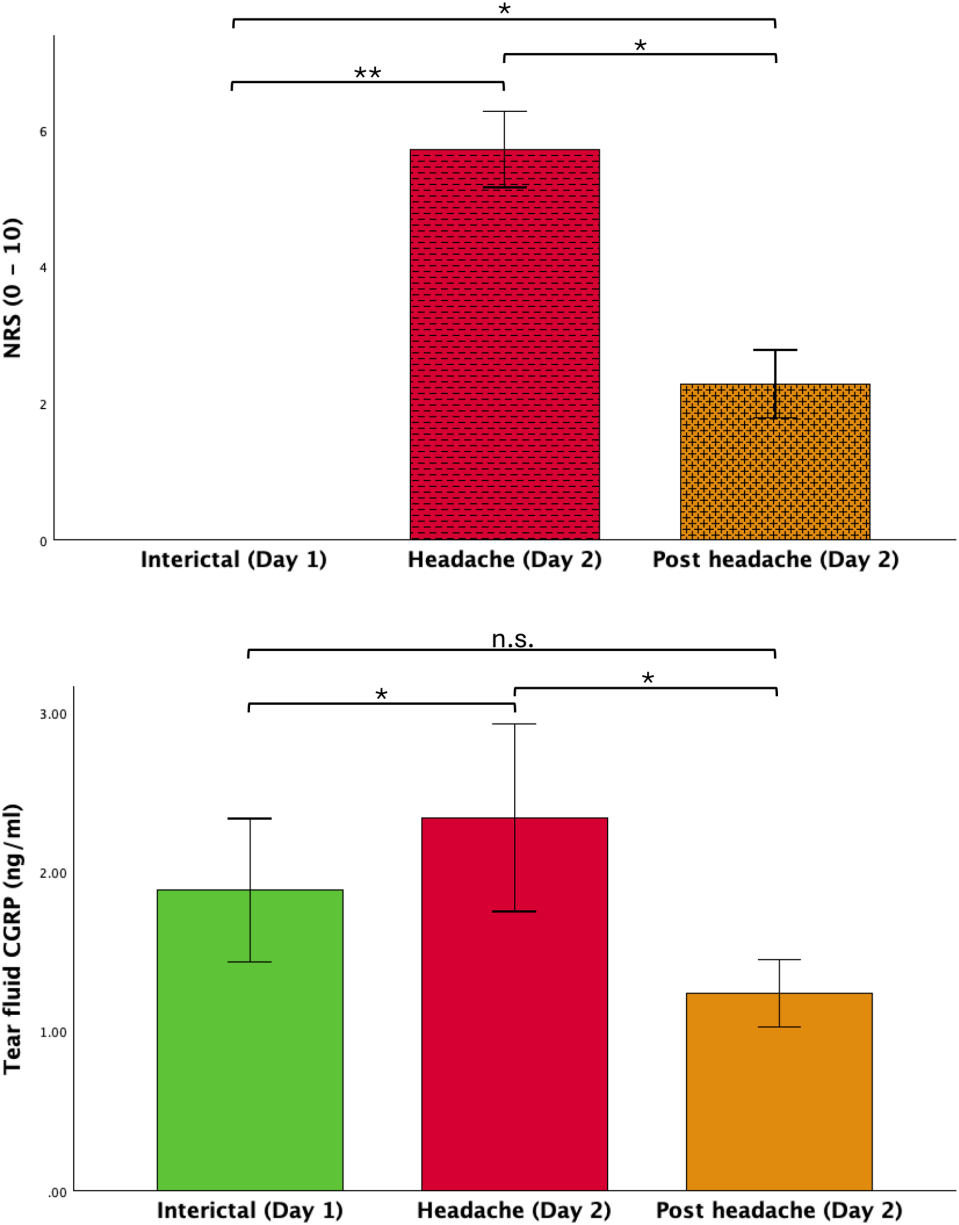
Headache intensity and tear fluid CGRP levels at baseline and during a spontaneous migraine attack** A** Migraine patients were investigated twice, interictally and during a spontaneous migraine attack. At study day 1 (‘interictal’), participants were headache- and medication-free for 48 h prior and after the study visit. During the spontaneous attack, migraine patients reported a maximum headache of 5.7 ± 2.1 on the NRS (‘headache’). Headache intensity improved after intake of acute medication in all participants and was significantly different between the timepoints (‘post headache’: 2.3 ± 1.8, *p* = 0.008). **B** Tear fluid CGRP was significantly higher during the spontaneous migraine attack on study day 2 (‘headache’: 2.34 ± 2.20 ng/ml) compared to interictal CGRP levels (‘interictal’: 1.89 ± 1.68 ng/ml) and after intake of acute medication and headache improvement (‘post headache’: 1.23 ± 0.80 ng/ml, *p* = 0.004), * *p* < 0.05, ** *p* < 0.001, n.s. not significant

Maximum headache intensity (‘headache’, NRS 5.7 ± 2.1) was significantly higher compared to interictal baseline (0 ± 0) and ‘post headache’ (2.3 ± 1.8; Friedman χ^2^ [2] = 26.528, *p* < 0.001, *n* = 14; post-hoc Dunn-Bonferroni test ‘interictal’ vs. ‘headache’ z = -1.893, *p* < 0.001; ‘headache’ vs. ‘post headache’ z = 0.893, *p* = 0.018; ‘interictal’ vs. ‘post headache’ z = -1.000, *p* = 0.008, Fig. 1A).

Tear fluid CGRP levels were significantly higher at maximum headache intensity compared to ‘interictal’ baseline as well as ‘post headache’ (‘interictal’: 1.89 ± 1.68 ng/ml, ‘headache’: 2.34 ± 2.20 ng/ml, ‘post headache’: 1.23 ± 0.80 ng/ml; Friedman χ^2^ [2] = 11. 286, *p* = 0.004, *n* = 14; post-hoc Dunn-Bonferroni test ‘interictal’ vs. ‘headache’ z = -0.929, *p* = 0.014; ‘headache’ vs. ‘post headache’ z = 1.214, *p* = 0.001; ‘interictal’ vs. ‘post headache’: z = 0.286, *p* = 1.000, Fig. 1B).

### Effect of time since attack onset

**Fig. 2:**
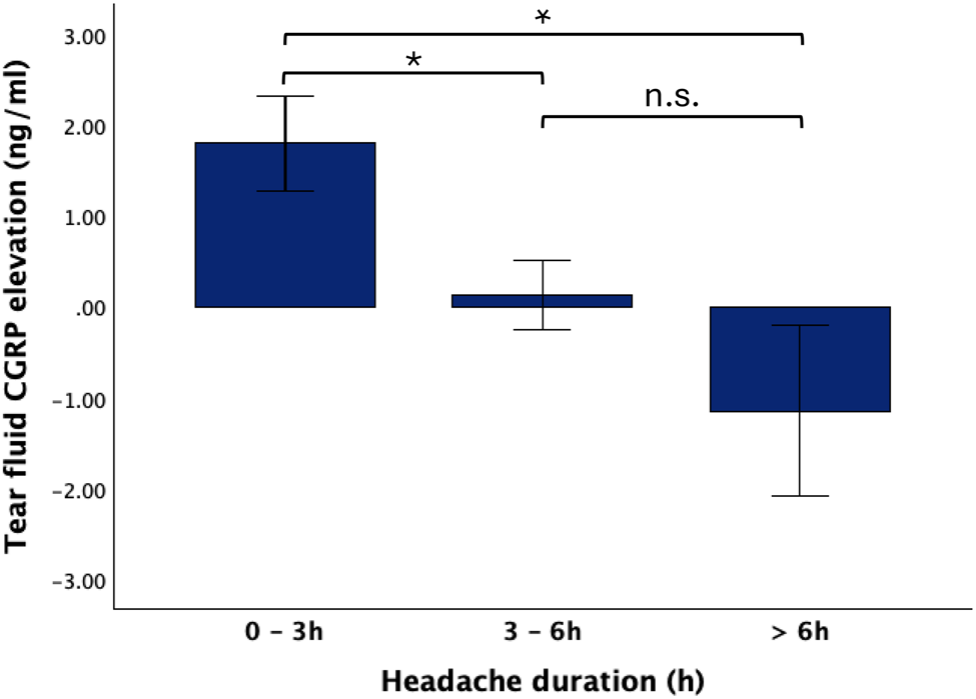
Participants arrived at the study center at different timepoints after onset of the migraine attack. Participants were grouped according to time since headache onset at the “headache” timepoint from 0–3 h, 3–6 h and > 6 h. Tear fluid CGRP levels were significantly more elevated (with respect to interictal levels) shortly after attack onset than later during the migraine attack (0–3 h: +1.80 ± 1.18 ng/ml, 3–6 h: +0.13 ± 0.93 ng/ml, > 6 h: -1.15 ± 1.63 ng/ml, *p* = 0.017), * *p* < 0.05, n.s. not significant

The extent of rise in tear fluid CGRP levels at the ‘headache’ timepoint compared to ‘interictal’ timepoint was significantly dependent on time since attack onset. Patients presenting to the study center with less than 3 h since attack onset showed a higher rise in tear fluid CGRP compared to patients with 3–6 h or more than 6 h since attack onset (0–3 h: +1.80 ± 1.18 ng/ml, n = 5, 3–6 h: +0.13 ± 0.93 ng/ml, n = 6; > 6 h: -1.15 ± 1.63 ng/ml, n = 3; Kruskal-Wallis-Test: U = 8.093, p = 0.017; post-hoc Dunn-Bonferroni test 0–3 h vs. 3–6 h: z = 5.600, p = 0.027; 0–3 h vs. > 6 h: z = 7.933, p = 0.009; 3–6 h vs. > 6 h: z = 2.333, p = 0.430, Fig. 2).

### Effect of type of abortive medication

All participants took acute medication. 4 patients (28.6%) took a triptan, 8 patients took a NSAID (57.1%), while 2 patients (14.3%) took a triptan and a NSAID. Only patients who took either a triptan or a NSAID, were included in the analysis. There was no significant difference in reduction of headache intensity from ‘headache’ to ‘post headache’ (NRS: triptan: − 4.1 ± 3.0, NSAID: − 3.1 ± 2.1; Mann-Whitney-U-Test: U = 20.500, *p* = 0.461) or reduction of tear fluid CGRP levels in patients taking triptans vs. NSAIDs (triptan: -0.58 ± 0.54 ng/ml, NSAID: -1.34 ± 2.26 ng/ml; Mann-Whitney-U-Test: U = 15.000, *p* = 0.933).

## Discussion

This study investigated tear fluid CGRP levels during spontaneous migraine attacks. Our results show that tear fluid CGRP rises significantly during spontaneous migraine attacks. Further, our results suggest that the elevation of CGRP is more pronounced at the beginning of a migraine attack. To the best of our knowledge, this is the first study that shows elevated tear fluid CGRP levels during spontaneous migraine attacks. Our results are supported by earlier studies finding elevated CGRP levels in blood and saliva, although to date CGRP has been rarely investigated in spontaneous migraine attacks [9, 14–16].

The first description of elevated ictal CGRP levels was in jugular vein blood of 8 migraine patients during spontaneous migraine attacks lasting between 2 h and 3 weeks [15]. In addition, it was shown that CGRP levels were significantly reduced after administration of 3–6 mg subcutaneous sumatriptan.

Two further studies detected elevated CGRP levels during spontaneous migraine attacks in the external jugular vein and antecubital vein, respectively [14, 16]. Both studies used radioimmunoassays to detect CGRP. Forty-five migraine patients were examined interictally in comparison to 20 healthy control subjects and ictally during spontaneous migraine attacks [14]. CGRP was detected in peripheral blood. It was shown that there was no significant difference in CGRP levels between migraine patients and control subjects during the interictal period. The ictal CGRP levels were significantly elevated compared to the interictal CGRP levels. Furthermore, it was shown that migraine patients who were examined within 2 h of the onset of the migraine attack showed highest CGRP levels [14].

In another study, CGRP levels were examined in 20 episodic migraine patients during a spontaneous migraine attack in relation to their response to rizatriptan [16]. The patients presented within 2 h of the onset of the migraine attack, and CGRP levels were determined in external jugular vein blood. It was shown that ictal CGRP levels were significantly higher in rizatriptan responders than non-responders. After taking rizatriptan, CGRP levels were significantly reduced in responders after 1 h and remained low for 12 h. Non-responders showed no reduction in CGRP levels. The authors concluded that increased trigeminal activation is associated with a better response to triptans. Interictal CGRP levels were not determined in this study, so a comparison between interictal and ictal levels cannot be made.

The results of CGRP testing in blood are inconsistent, so other detection methods have been evaluated [6, 7]. In one study, CGRP levels were examined in saliva of 22 episodic migraine patients and 22 healthy control subjects [9]. A special feature of this study was that the participants took their own saliva samples interictally and during a spontaneous migraine attack. The study showed elevated interictal CGRP levels in migraine patients compared to healthy controls. Ictal saliva CGRP levels were elevated compared to interictal CGRP levels, and were significantly higher early during the headache attack, similar to our findings.

From our study results and the above-mentioned studies, it can be concluded that CGRP is a marker for trigeminal activation and, due to its early increase, plays an important role in the initiation of migraine attacks.

The evidence of ictal increase in CGRP is a further step in the validation of the tear fluid CGRP as a biomarker for migraine attacks, as elevated CGRP levels have now been shown during both spontaneous and experimentally induced migraine attacks [11]. In a further study with a larger number of subjects, the investigation of tear fluid CGRP levels dependent on the headache severity would be interesting as it has been shown earlier that more severe headache attacks are associated with a greater increase in CGRP [11]. The present and previous data already provide evidence that there is a strong increase in the peptide early during the attack, but it would be interesting to measure different time points over the course of the attack. In the present study, all study participants took acute medication relatively early after collection of the first ictal samples, so the spontaneous course cannot be described.

However, we see a significant reduction of pain intensity and of CGRP levels after taking acute medication, which is further evidence of the role of CGRP as a trigeminal marker. Similarly, earlier studies showed a reduction of CGRP after intake of triptans [15, 16]. Here, we were able to demonstrate that tear fluid CGRP levels are also reduced after intake of NSAIDs, which matches our earlier findings in experimental migraine attacks [11].

Detection of the peptide in tear fluid is feasible, as it has been demonstrated in previous studies. The advantages of measuring tear fluid include ease of application, as demonstrated by ictal and interictal measurements. Further, measuring close to the site of peptide release appears to be an advantage due to higher peptide concentrations and less peptide degradation [8, 17].

### Limitations

A significant limitation of the study is the small number of participants, which meant that subgroup analyses was not possible. The study was conducted during the restrictions imposed by the COVID-19 pandemic, resulting in only 16 of the initial 82 patients being studied during a spontaneous migraine attack.

In addition, it would have been interesting to include all subjects at an earlier stage of the migraine attack, but this was not possible due to work commitments or other obligations of the participants. In this study, the Cusabio^®^ ELISA was used to detect CGRP.

It has been suggested that this ELISA does not detect CGRP [18]. However, in a previous study, we were able to show that the tear fluid CGRP levels show similar results using the Cusabio^®^ and Abbexa^®^ ELISA, so we believe that the study results are robust [11].

## Conclusions

Detection of CGRP in tear fluid is feasible and elevated tear fluid CGRP levels during spontaneous migraine attacks underline the role of CGRP in migraine pathophysiology. The higher elevation of tear fluid CGRP at the beginning of the migraine attack might be the reason why abortive medication is more effective if used early. This study corroborates CGRP as a marker of trigeminal activation.

## Data Availability

All data supporting the findings of this study are available within the paper .
